# Supplementary material for: Endogenous tenocyte activation underlies the regenerative capacity of the adult zebrafish tendon
Source: NPJ Regen Med. 2023 Sep 19;8:52. doi: 10.1038/s41536-023-00328-w (PMC10509205; doi:10.1038/s41536-023-00328-w)
Supplement: Supplementary file 1 — Supplementary Materials [file 41536_2023_328_MOESM1_ESM.pdf]

Supplementary figures and legends

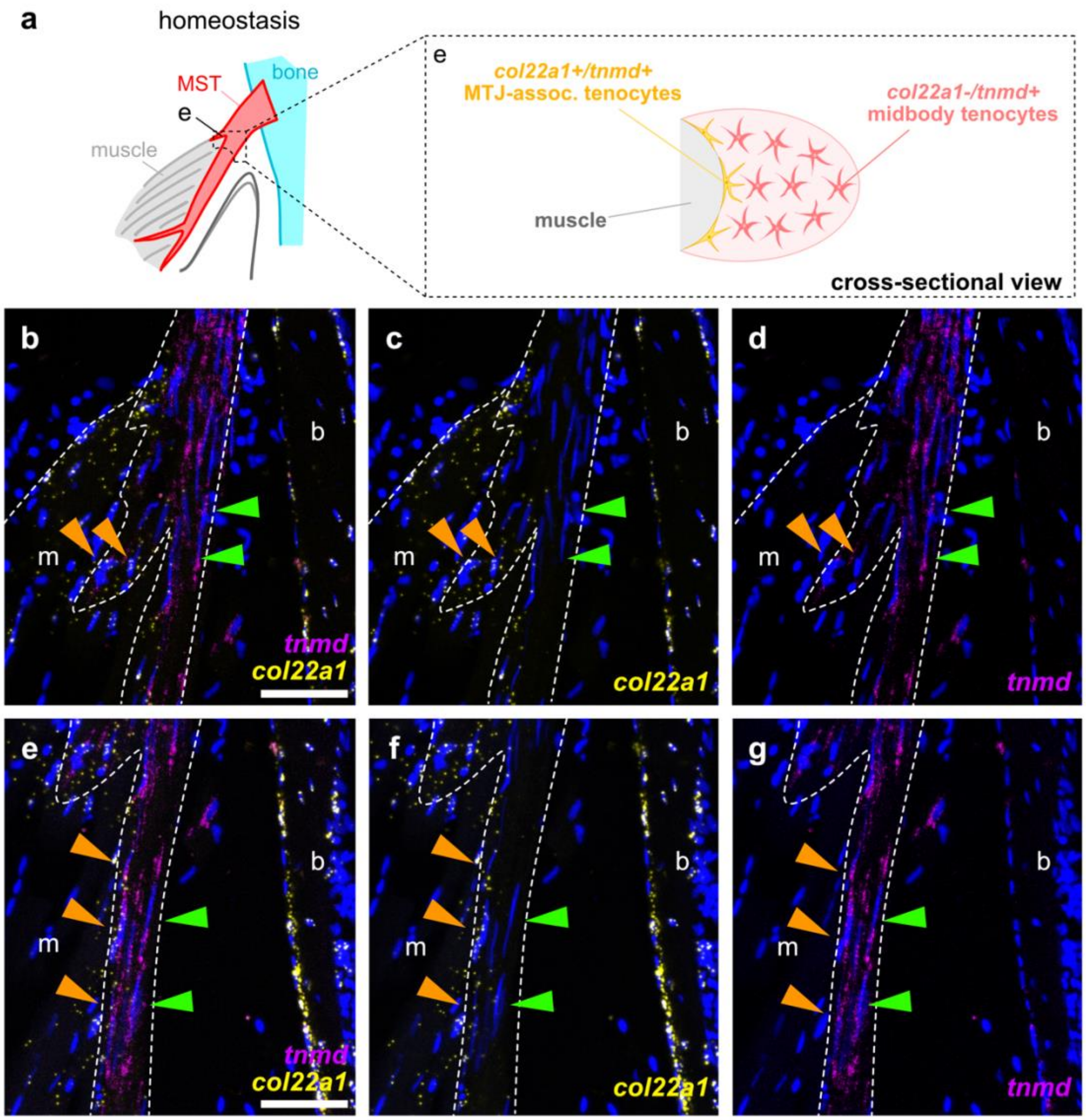

Supplementary Figure 1. The MST contains cells expressing midbody tenocyte and myotendinous junction markers.

(a) Graphical schematic showing a cross-sectional view of the MST during homeostasis to depict the cellular composition based on the expression patterns shown in e.

(b-g) Section RNAscope *in situ* hybridization of *tnmd* and *col22a1* on an uninjured adult control MST showing expression along two different regions of the tendon. Panels b-d show a region at the beginning of the MTJ and the midbody/MTJ section. Panels e-g show the section of the

midbody that contains MTJ cells along the length of the tendon. *Col22a1* expression is specifically localized to the tendon cells that are in direct contact (*tnmd+/col22a1+*) with the muscle (m) (orange arrowheads), while *tnmd+*/*col22a1-* cells are present in tenocytes not directly in contact with the muscle along the length of the tendon (green arrowheads). *Col22a1+* cells can also be seen in periosteal cells lining the maxillary bone (b).

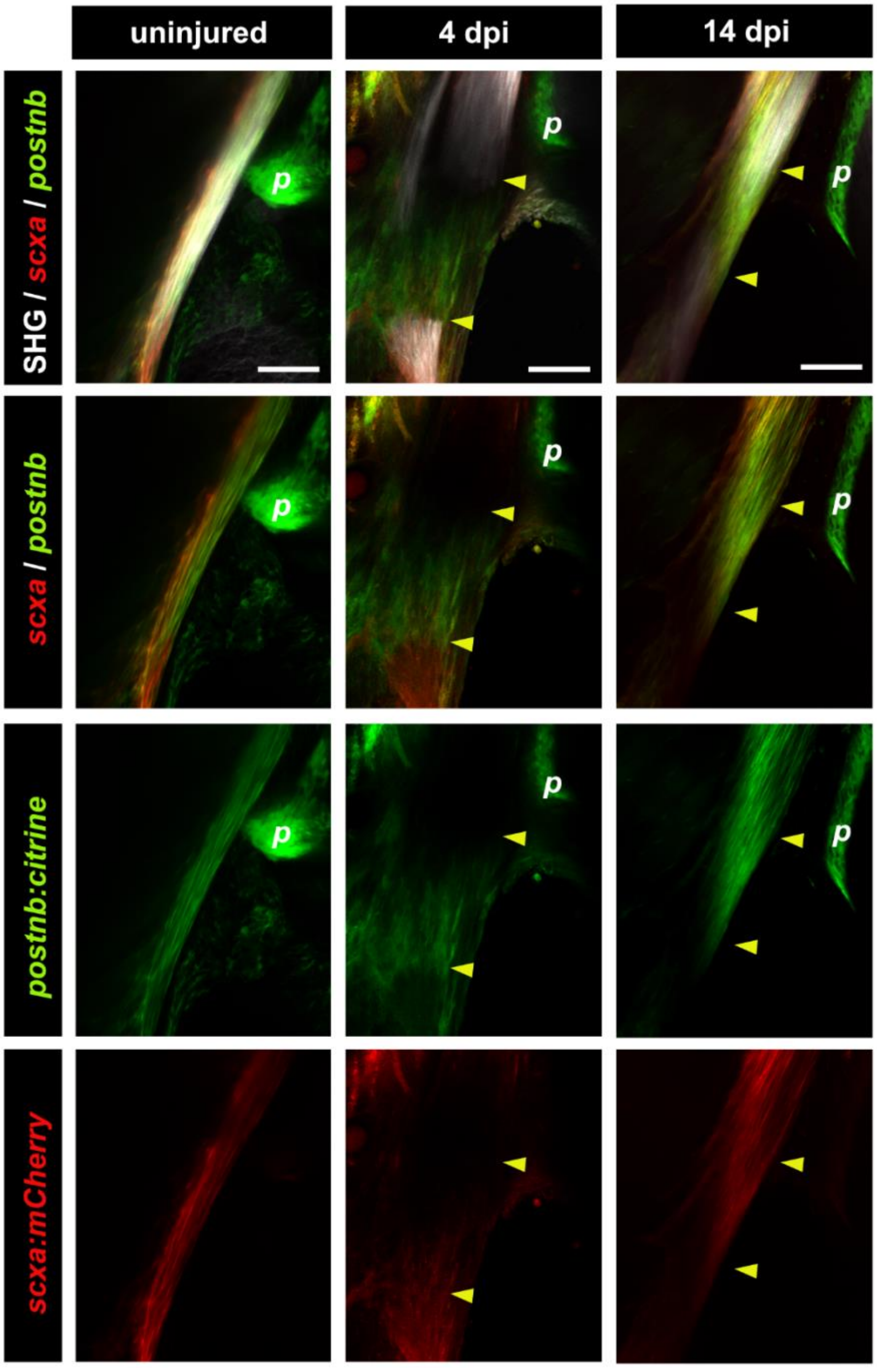

Supplementary Figure 2. *Postnb:citrine* expression during tendon regeneration. Z-projections of sub-slices from 2-photon stacks of uninjured and regenerating (4 and 14 days post-injury (dpi)) MSTs in *postnb:citrine;scxa:mCherry* zebrafish overlaid with second harmonic generation (SHG) signal. Yellow arrowheads denote the severed tendon ends and the boundaries of the defect. p, periosteum. Scale bar, 100 µm.

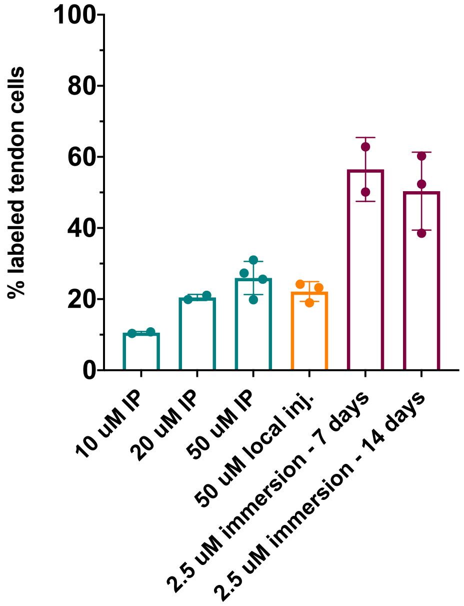

Supplementary Figure 3. Optimizing 4OH-T delivery to obtain maximal tendon labeling during homeostasis. Quantification of the percentage of CFP+ and/or YFP+ labeling in *scxa:creERT2;ubi:zebrabow* zebrafish after various methods of 4OH-T delivery. For the IP injections, ~10 µL of 4OH-T at the various concentrations detailed was injected daily for 3 consecutive days. For the local injection, ~10 µL 50 µM of 4OH-T was injected locally into the cavity between the skin and the jaw adductor muscle attached to the MST. For immersion, zebrafish were incubated for 3 nights in 2.5 µM of 4OH-T. For all conditions, the labeling was analyzed 7 days after the last treatment, except for immersion which was assessed at both 7 and 14 days post-induction. IP, intraperitoneal; dps, days post-induction. Each point represents a biological replicate from a different fish and the error bars depict mean ± standard deviation.

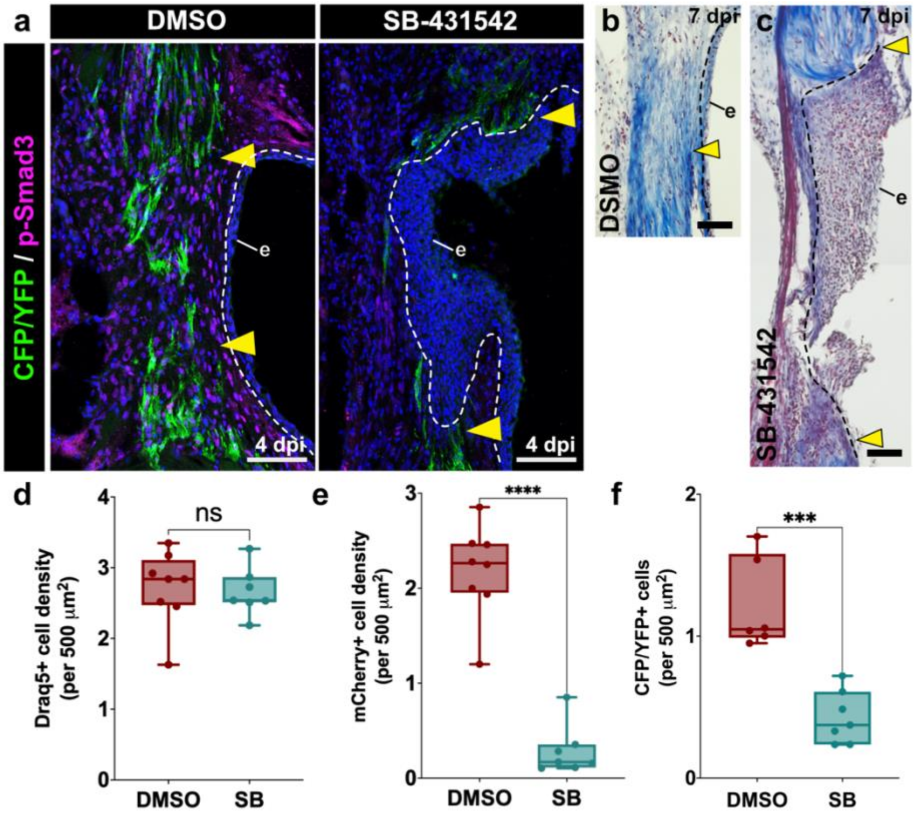

Supplementary Figure 4. Validation of TGF-β inhibition via SB-431542 treatment.

(a) Double immunostaining of CFP+ and/or YFP+ *scxa*-lineage cells (in green) with p-Smad3 (in magenta) at 4 days post-injury (dpi). Yellow arrowheads denote the boundary of the severed tendon ends. Scale bar, 100 µm.

(b-c) Masson's trichrome staining of DMSO- or SB-431542-treated tendons at 7 dpi when treated from 1-7 dpi. Yellow arrowheads denote the boundary of the severed tendon ends (Note: top stub was not captured in section shown in b). Black dotted lines denote the boundary of the epithelium. e, epithelial cells. Scale bar, 20 µm.

(d-f) Quantification of general cell density (d), *scxa:mCherry*+ cell density (e), and *scxa*-lineage cell density (f) in the injury site of regenerating tendons at 7 dpi in DMSO- and SB-431532 (SB) treated zebrafish (excluding skin) following a 1-7 dpi treatment. Unpaired two-tailed t-tests were performed between DMSO and SB-431542 conditions for statistical analysis (d-e: DMSO, N=8,

SB, N=7 in *scxa:mCherry* line; f: DMSO, N=6, SB, N=7 in *scxa:creERT2*;*ubi:zebrabow* line).

****$p < 0.0001$, ***$p < 0.001$, ns, not significant.
